# Acute effect of short foot exercise on dynamic stability and foot kinematic in trail runners: a proof-of-concept study

**DOI:** 10.7717/peerj.20364

**Published:** 2025-11-17

**Authors:** Claudio Aguilar-Risco, Mauricio San Martín-Correa, Felipe Araya-Quintanilla, Joaquín Calatayud, Guillermo Mendez-Rebolledo

**Affiliations:** 1 Master in Health and Sports Sciences, Faculty of Medicine, Finis Terrae University, Santiago, Chile; 2 Escuela de Kinesiología, Facultad de Odontología y Ciencias de la Rehabilitación, Universidad San Sebastián, Valdivia, Chile; 3 Escuela de Kinesiología, Instituto de Aparato Locomotor y Rehabilitación, Universidad Austral de Chile, Valdivia, Chile; 4 Escuela de Kinesiología, Facultad de Odontología y Ciencias de la Rehabilitación, Universidad San Sebastián, Santiago, Chile; 5 Exercise Intervention for Health Research Group (EXINH-RG), Department of Physiotherapy, Universidad de Valencia, Valencia, Spain; 6 Laboratorio de Investigación Somatosensorial y Motora, Escuela de Kinesiología, Faculta de Salud, Universidad Santo Tomás, Talca, Chile

**Keywords:** Short foot exercise, Intrinsic foot muscle, Arch height index, Trail running, Training volume, Dynamic balance

## Abstract

**Background:**

Trail runners face uneven terrains requiring optimal foot stability and postural control. The short foot exercise (SFE) may acutely enhance dynamic balance and foot arch height, potentially mitigating injury risk. This study aimed to evaluate the acute effects of the SFE on postural control and kinematics during a dynamic balance test in trail runners, considering the variations in the weekly training volumes of the participants.

**Methods:**

Sixteen adult trail runners (mean age 36 ± 8.4 years; 50% male) with at least one year of trail running experience were evaluated. Dynamic balance was assessed using the Y-Balance Test (YBT), and kinematics of the foot arch were measured via the Arch Height Index (AHI) using a 3D motion capture system. Baseline measurements were taken, followed by an SFE protocol: the participants had to perform 12 repetitions of 5-second contractions, which they did in three sets with 2 minutes of rest between sets. Immediately afterward, YBT and AHI were reassessed.

**Results:**

YBT showed significant improvements in all reach directions after the application of the SFE (*p* < 0.05). In contrast, no significant changes were observed in the AHI across reach directions (*p* > 0.05). Yet, subgroup analysis by weekly training volume revealed that participants with higher weekly volumes experienced a significant increase in anterior AHI (mean difference = −0.54 mm; 95% CI [−1.09 to 0.01]; *p* = 0.027; effect size = 0.13). The SFE may acutely improve foot kinematics and dynamic balance in trail runners; however, these effects are influenced by the weekly training volume of the participants.

**Conclusion:**

This study suggests that a single session of the SFE may induce acute improvements in arch height and dynamic balance in trail runners, with differential responses depending on training volume. These preliminary results highlight the potential of the SFE as an acute activation strategy for the intrinsic foot muscles, but caution should be exercised when extrapolating the results.

## Introduction

Trail running is an outdoor running discipline performed across diverse terrains, including mountains, deserts, native forests, tropical jungles, coastal areas, grassy plains, and arid regions (Scheer et al., 2020). In recent years, this sport has experienced remarkable growth in popularity, has been growing at a global rate of 12% yearly for the last decade (Rosenkrantz, Schuurman & Lear, 2024), establishing itself as the leading all-terrain running discipline (Mocanu, 2015). Trail runners face various challenges, including significant elevation changes, diverse environmental and weather conditions, and uneven terrains (Mocanu, 2015; Scheer et al., 2020). While the physical health benefits of running are well-documented, navigating trails with heterogeneous surfaces increases the risk of injury (Matos et al., 2021; Viljoen et al., 2021). The foot-ankle complex is among the most frequently injured regions of the body, accounting for approximately 50% of injuries, characterized by ligament, or joint capsule injuries (Viljoen et al., 2021; Viljoen et al., 2022). In this sense, training workloads are also central to the development of injuries for runners (Drew & Finch, 2016). It has been proposed that higher training loads lead to higher injury rates (Gabbett, 2016; Soligard et al., 2016), and if these weekly training workloads increase rapidly, the athlete faces a high risk of injury (Gabbett et al., 2016). Training for more than 5.4 h per week, including competition hours, is associated with a higher risk of injury in individual sports (Hartwig et al., 2019). Furthermore, in team sports, an additional hour of weekly training has been found to increase the risk of injuries in young athletes, with ankle sprains being among the most common injuries (Giroto et al., 2017). However, if training workloads are applied progressively, they can foster resilience in athletes, resulting in lower injury rates and performance optimization (Gabbett, 2016).

Trail running requires quick cognitive processing of the environment, the ability to respond to variations in the ground surface, obstacle avoidance, and positional adjustments of the foot in response to the terrain’s elevations (Vincent, Brownstein & Vincent, 2022). To maintain postural control in this type of activity, runners use anticipatory and reactive strategies in response to obstacles on the roads (Vincent, Brownstein & Vincent, 2022). Previous research has shown that running on uneven surfaces significantly alters lower-limb biomechanics compared to smooth terrain. Specifically, running on uneven surfaces increases energy expenditure by approximately 5%, elevates stride width and length variability by about 25%, and increases leg stiffness by around 20%, all of which indicate greater demands on postural control (Voloshina & Ferris, 2015). Furthermore, increased hip and knee flexion has been observed on uneven terrain, suggesting a shift toward postural stability rather than segmental stiffness (Ivaniski-Mello et al., 2024). These biomechanical challenges, particularly relevant for trail runners navigating unpredictable terrain, reinforce the need to explore strategies aimed at improving neuromuscular function and balance in this population. The short foot exercise (SFE) has been used to improve postural control in various demanding motor tasks by contracting and activating the intrinsic foot muscles (IFM) (Moon & Jung, 2021). The IFM are a subset of the central foot system that play an important role in static and dynamic posture, helping to improve movement control, stabilization, and foot flexibility during stance phase. They allow for the absorption of impact energy, enhance the dynamic alignment of the foot, support the medial longitudinal arch (MLA) of the foot, and provide plantar proprioceptive feedback (Kelly et al., 2014; McKeon et al., 2015). The most important element of foot function is the MLA, as postural demands increase, the IFM stabilize the foot, directly improving the posture of the foot arch *via* significant changes in the length and height of the MLA (Kelly et al., 2012; Kelly et al., 2014; Mulligan & Cook, 2013). These foot arch changes can be compared through validated clinical kinematic methods such as the arch height index (AHI), which is used to evaluate changes in MLA in static and dynamic tasks (Uhan et al., 2023). In recent years, it has been demonstrated that foot training with the SFE provides benefits in static and dynamic postural control in healthy young individuals, people with flat feet, and even those with ankle instability. The SFE improves the muscle strength of the IFM, consequently, achieving adequate support of the medial longitudinal arch (Lynn, Padilla & Tsang, 2012; Mulligan & Cook, 2013; Kim & Kim, 2016; Lee & Choi, 2019; Moon & Jung, 2021). Moreover, it has been demonstrated that training this musculature reduces the risk of injury by 2.42 times compared to a control group of recreational runners (Suda et al., 2022). The authors of this study point out that with greater foot strength, the runner may sustain longer distances without developing a running-related injury, as the SFE acts as a protective factor against lower limb injuries. To the best of our knowledge, no studies have explored the benefits of strengthening the intrinsic foot musculature in trail runners.

Foot stability, directly modulated by the intrinsic foot muscle, may play an important role in motor tasks requiring dynamic postural control or movements that demand more effort, such as climbing slopes, accelerating, decelerating, or jumping (Besomi et al., 2018). However, the potential acute effects of the short foot exercise on dynamic postural control and kinematics of the foot arch have not yet been investigated as a possible strategy for injury prevention in trail runners. Furthermore, the influence of weekly training volume on foot stability and kinematics in trail runners remains unknown. While high training volumes have been associated with an increased risk of injury when applied excessively or without appropriate progression (Gabbett, 2016; Hartwig et al., 2019) progressive and chronic exposure to mechanical load can also lead to beneficial neuromuscular adaptations in trained individuals. High-weekly-mileage runners have demonstrated more efficient landing strategies and improved joint stiffness regulation compared to low-weekly-mileage runners, suggesting enhanced neuromuscular control during dynamic tasks (Verheul, Clansey & Lake, 2017). Additionally, targeted training of the intrinsic foot muscles has been shown to increase muscle volume and improve running mechanics in recreational runners (Taddei et al., 2020). Therefore, this study aimed to evaluate the acute effects of the short foot exercise on postural control and kinematics of the foot arch during a dynamic balance test in trail runners, considering the participants’ varying weekly training volumes. We hypothesize that the short foot exercise will immediately enhance dynamic balance and increase the arch height index of the foot in trail runners. In addition, we anticipate that runners with higher weekly training volumes will exhibit greater improvements in arch height index and dynamic balance compared to those with lower training volumes.

## Material & Methods

### Study design

This was a proof-of-concept study. Dynamic balance was assessed before and after the application of the short foot exercise using the Y-Balance Test (YBT) in the anterior, posterolateral, and posteromedial directions. In addition, kinematics of the foot arch were evaluated through the arch height index during the YBT performance.

### Participants

A non-probabilistic convenience sampling was conducted, resulting in a sample of 16 volunteers. The sample size was determined using G*Power software (version 3.1.9.7; Franz Faul, University of Kiel, Kiel, Germany) based on an effect size of 0.66 in the dynamic balance outcome following SFE, an alpha error of 0.05, and a power of 0.80, which were calculated from data from a previous study (Lynn, Padilla & Tsang, 2012). To recruit participants, every running club in the city of Valdivia (Chile) was contacted by sending a form *via* email to identify potential participants. The club members answered questions regarding the study’s inclusion and exclusion criteria. Those who met the criteria were invited by phone call to participate in the study. The study included trail runners who were 18 years old or older, both female and male, who were registered and active in an official running club in Valdivia, with at least 1 year of experience in trail running, and who had completed a trail event of 10 km or more in the last year. In addition, they had to accumulate a weekly distance volume of 20 km or more, with a training frequency of at least three times per week and a total of four hours of training per week (Besomi et al., 2018; Suda et al., 2022). Participants with functional lower limb injuries that prevented them from practicing trail running, such as fractures or ligament injuries, were excluded. Also individuals undergoing physical therapy for strengthening the intrinsic foot muscles and ankle, or for specific balance rehabilitation, as well as those wearing minimalist footwear, were also excluded (Lynn, Padilla & Tsang, 2012; Suda et al., 2022).

### Ethical approval

All participants received written and oral information about the project and gave informed consent, consistent with the Helsinki Declaration guidelines before participating in the study. The research protocol received approval from the Health Service Ethics Committee, Valdivia, Chile (No 159).

### Procedures

Participants were invited to the Human Movement Analysis Laboratory at the School of Kinesiology of the Universidad Austral de Chile. Before beginning the study, the principal investigator showed and explained the SFE and the YBT. The participants practiced familiarizing themselves with the study protocol. Data such as sex, age, body mass, height, body mass index (BMI), years of running experience, weekly training volume (hours per day of training), and weekly accumulated kilometers were recorded on a data sheet. The study, conducted in a single session lasting approximately 2 h, began with measurements of full leg length, measured between the anterior superior iliac spine (ASIS) and the medial malleolus, *via* the knee joint, the medio-lateral width of the knees across the line of the knee axis and the medio-lateral distance across the malleoli. Reflective markers were then placed on the ankle-foot segment to evaluate kinematics using infrared cameras (VICON model T10-S; Vicon, Oxford, UK). These are used to calculate joint center positions and must be measured and entered into software VICON before any modeling can take place can begin. Subsequently, the participants performed the YBT before the foot intervention. As soon as the initial YBT evaluation was completed, the participants immediately performed the SFE protocol. At the end of the SFE protocol, the YBT was immediately performed again to assess the dependent variables (AHI, YBT).

#### Arch Height Index

**Figure 1:**
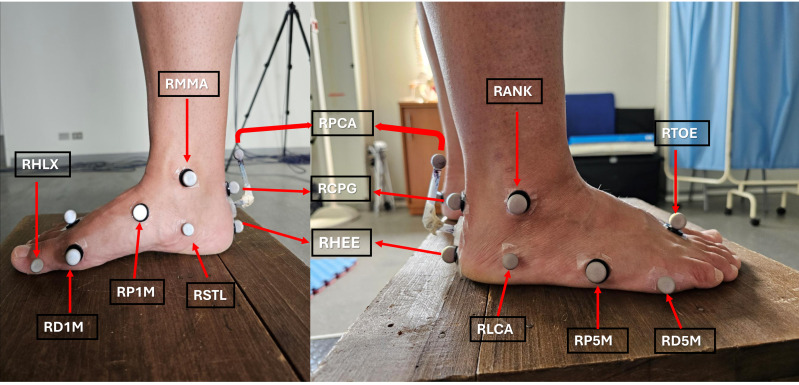
(Left) Markers on the foot shown from a medial view. (Right) Markers on the foot and ankle shown from a lateral view. RMMA, Medial Malleoli; RHLX, Hallux; RD1M, 1st Metatarsal, distal medial; RP1M, 1st Metatarsal, proximal medial; RSTL, Sustaniculum Tali; RHEE, Heel; RCPG, Posterior end of the calcaneus; RPCA, Posterior calcaneus proximal; RLCA, Lateral calcaneus; RP5M, 5th Metatarsal, proximal lateral; RD5M, 5th Metatarsal, distal medial; RTOE, 2nd Finger base; RANK, lateral malleolus.

The AHI was computed using kinematic data collected with the VICON 3D motion capture system, equipped with six infrared cameras operating at a capture frequency of 120 Hz. Reflective markers were attached to the skin using double-sided adhesive tape and placed on specific bony landmarks, as illustrated in Fig. 1. This setup enabled digital reconstruction and kinematic modeling of the ankle and foot using the Oxford Foot Model (OFM) protocol (Merker et al., 2015). In accordance with the VICON Plug-in Gait and OFM documentation, the AHI was defined as the ratio between the perpendicular distance of the RP1M marker (first metatarsal, proximal medial) from the plane defined by the RD1M (first metatarsal, distal medial), RP5M (fifth metatarsal, proximal lateral), and RD5M (fifth metatarsal, distal medial) markers, and the foot length, measured as the linear distance from the RHEE (heel) marker to the RTOE (second finger base) marker. This calculation reflects the medial longitudinal arch height normalized to foot size and was performed for each trial during the dynamic balance task. Three trials per condition were averaged for analysis. This method has been shown to be reliable for assessing foot arch behavior under dynamic conditions (Carson et al., 2001; Stebbins et al., 2006). Moreover, the AHI represents the kinematic changes that the medial longitudinal arch (MLA) may undergo during motor tasks (Uhan et al., 2023).

#### Y-Balance Test

Dynamic balance was assessed using the YBT which is a clinical adaptation of the Star Excursion Balance Test (SEBT) (with an intrarater reliability coefficient ranging from 0.85 to 0.91) (Plisky et al., 2024). A “Y” shape was marked on the laboratory floor using adhesive tape. Participants were barefoot and positioned the toes of their dominant foot (defined as the foot used to kick a ball) at the center of the Y. This leg maintained balance while the opposite leg reached as far as possible in three directions: anterior, posteromedial, and posterolateral. Skill cones were placed at the starting point of each direction, which participants were instructed to push using the reaching foot, aiming to achieve the maximum possible distance. The distance reached was measured with a measuring tape, based on the final position of the displaced cone. Prior to the main testing, participants completed six practice trials in each direction to familiarize themselves with the procedure (Plisky et al., 2009). To enhance reproducibility and ensure consistency, a standardized testing order was followed: anterior reach first, followed by posterolateral, and finally posteromedial. This sequence was repeated three times per direction (Plisky et al., 2009). A trial was considered invalid and had to be repeated if the participant failed to maintain unipedal stance, lost contact between the reaching foot and the cone, used the cone for balance support, or was unable to return the reaching foot to the starting position (control position) (Plisky et al., 2024). Once each round was completed, the distance reached was recorded, and results were normalized using the following formula:

\begin{eqnarray*}\%~Normalized~reach~distance= \frac{reach~distance~ \left( cm \right) }{leg~length~(cm)} \times 100. \end{eqnarray*}

#### Short Foot Exercise

**Figure 2:**
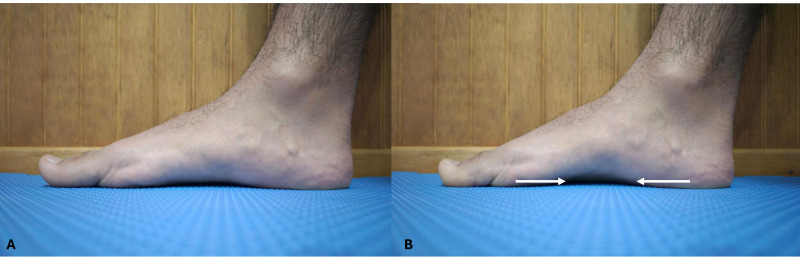
(A) Starting position with the foot at rest. (B) Performing the short-foot exercise. The arrows indicate the direction in which the participant should contract the muscles by drawing the metatarsal heads toward the heel and the heel toward the metatarsal.

The SFE is an exercise that involves contraction of the midfoot by drawing the metatarsal heads posteriorly toward the calcaneus without flexing the toes, resulting in elevation of the medial longitudinal arch (McKeon et al., 2015; Jaffri et al., 2023). The goal is to selectively activate the intrinsic foot muscles, particularly the lumbricals and interossei, by promoting flexion at the metatarsophalangeal and proximal interphalangeal joints, while minimizing the contribution of extrinsic muscles acting on the distal interphalangeal joint during balance control (Jastifer, 2023). Participants were first familiarized with the SFE to ensure correct performance and awareness of the specific muscle activation required. The exercise was performed in a seated position with feet flat on the ground. Participants were instructed to perform a voluntary and conscious maximal contraction of the intrinsic musculature of the dominant foot. The verbal cue used was to “draw the metatarsal heads toward the heel and the heel toward the metatarsal heads” without generating flexion of the metatarsophalangeal joints (see Fig. 2 for reference). The SFE protocol was administered immediately after the initial YBT assessment. Participants performed 12 repetitions of 5-second maximal voluntary contractions, structured into three sets with 2-minute rest intervals between sets, following a protocol of previous studies (Moon, Kim & Lee, 2014; Lee, Cho & Lee, 2019). Immediately after completing the SFE protocol, the YBT was re-administered to assess the post-intervention changes in dynamic balance performance.

### Statistical analysis

The total sample was described in terms of sex, age, body mass, height, BMI, accumulated kilometers, years of practice, and weekly training volume, using percentages, frequencies, and standard deviations. Normality and homogeneity of variance were assessed using the Shapiro–Wilk and Levene’s tests, respectively. The paired sample *t*-test was used to compare pre and post the application of the SFE Subsequently, a two-factor repeated measures analysis of variance (ANOVA) (time: pre and post SFE application; training volume: low and high) was performed. Participants who practiced less than 5.4 h of training per week were considered to have a low training volume, while those who practiced 5.4 h or more (Hartwig et al., 2019) were considered to have a high training volume. Consequently, four groups were created for analysis. Multiple pairwise comparisons were applied using Bonferroni-corrected t-tests to determine if there were significant interactions between factors. Additionally, the effect size (ES) was calculated based on Cohen’s d and was reported as no effect (0 to 0.19), small effect (0.20 to 0.49), moderate effect (0.50 to 0.79), or large effect (0.80 or greater) (Fritz, Morris & Richler, 2012). For all analyses, a *p*-value <0.05 was utilized. IBM SPSS software version 29.0.1.0 for Windows (SPSS Inc, Chicago, IL, USA) was used for statistical analysis, while GraphPad software version (10.2.1.395) was used for graphing the results.

## Results

Table 1 presents the demographics, anthropometrics, and running variables of the study sample. Study sample characteristics were also divided according to the training volume of the participants.

**Table 1:** Demographic characteristics, anthropometry and variables associated with the practice of trail running (*α*).

Variables	Trail runners (*n* = 16)	Low training volume (*n* = 9)	High training volume (*n* = 7)
Female (*n*, %)	8 (50)	4 (44.4)	4 (57.1)
Age (year)	36 ± 8.4	38 ± 9.5	32.4 ± 6.5
Size (cm)	165.7 ± 7.1	165 ± 5.7	166.6 ± 9.0
Body mass (kg)	66 ± 7.8	66.4 ± 7.26	65.3 ± 9.0
Body mass index (kg/m^2^)	24.0 ± 1.7	24.3 ± 1.65	23.5 ± 1.7
Training volume (h × Training days/Week)	6.0 ± 1.9	4.7 ± 0.61	7.7 ± 1.5
Practice years (years)	3.5 ± 2.7	4.0 ± 3.07	2.9 ± 2.3
Weekly kilometers (km/week)	53.1 ± 17.7	47.8 ± 19.1	60 ± 14.1

**Notes.**

(*α*) Values are presented by means, ± (standard deviation) or percentages.

### Arch height index

**Figure 3:**
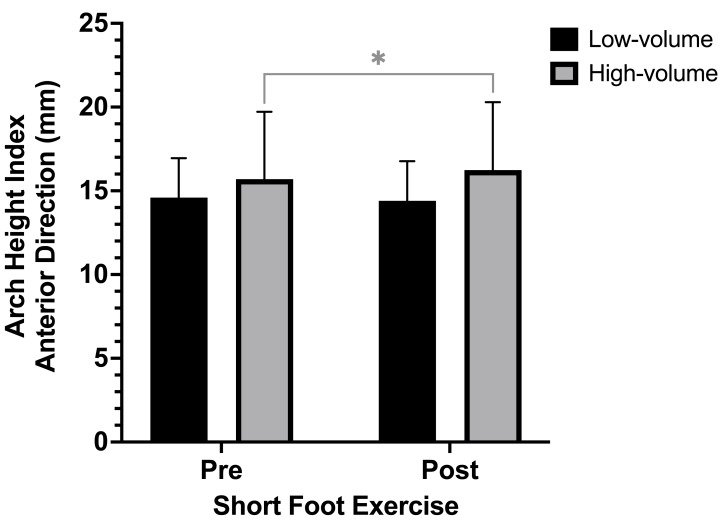
*Post-hoc* comparison of the AHI in the anterior reach of the Y-Balance Test between subjects with low and high volume training pre and post short foot exercises application. *p < 0.05.

There were no statistically significant differences pre and post-SFE in the anterior (mean difference (MD) = −0.17 mm; CI 95% [−0.59 to 0.26]; *p* = 0.199), posterolateral (MD = 0.02 mm; CI 95% [−0.76 to 0.79]; *p* = 0.480) and posteromedial (MD = 0.07 mm; CI 95% [−0.69 to 0.82]; *p* = 0.428) directions of the AHI of the foot. However, when categorizing by weekly training volume, ANOVA (pre and post-SFE x weekly training volume) revealed a significant interaction between the factors (*df* = 1; *F* = 8.232; *p* = 0.012) for the anterior reach direction of the AHI of the foot. The *post-hoc* analysis showed significant differences pre and post application of the SFE among participants with a high training volume (MD = −0.54 mm; CI 95% [−1.09 to 0.01]; *p* = 0.027; ES = 0.13) as seen in Fig. 3, while no significant differences were observed among participants with a low training volume. No significant differences were observed for the posterolateral and posteromedial reach directions of the AHI (*p* > 0.05).

### Y-Balance test

**Figure 4:**
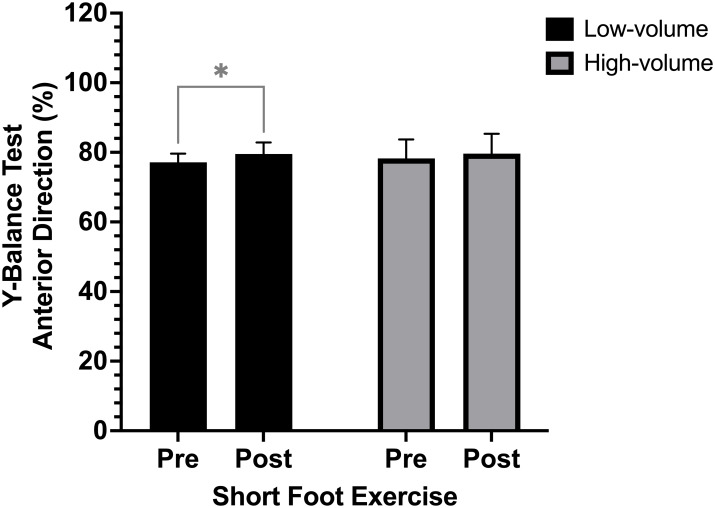
*Post-hoc* comparison of the percentage of reach in the anterior direction of the Y-Balance Test between subjects with low and high training volume pre and post short foot exercises application. *p < 0.05.

There were significant differences pre and post SFE application in the anterior (MD = −1.9%; CI 95% [−3.0 to −0.7]; *p* = 0.002; ES= 2.21) posterolateral (MD = −2.6%; CI 95% [−5.4 to 0.3]; *p* = 0.037; ES= 5.32) and posteromedial (MD = −5.2%; CI 95% [−8.3 to −2.0]; *p* = 0.002; ES= 5.88) directions of the YBT. Furthermore, when categorizing by weekly training volume, ANOVA (pre and post-SFE x weekly training volume) revealed a significant interaction between the factors (*df* = 1; *F* = 12.377; *p* = 0.03) for the anterior direction variable of the YBT. The *post-hoc* analysis showed significant differences pre and post application of the SFE among participants with a low training volume (MD = −2.4%; CI 95% [−4.4 to −0.4]; *p* = 0.018; ES= 0.09) as seen in Fig. 4, while no significant differences were observed among participants with a high training volume. No significant differences were observed for the posterolateral and posteromedial reach directions of the YBT (*p* > 0.05).

## Discussion

In the present study, we investigated the acute effect of SFE on dynamic balance and kinematics of the foot arch among trail runners, as well as the influence of weekly training volume on the study variables. There were significant differences pre and post SFE application in the all directions of the YBT. Besides, a significant improvement in the AHI of the foot was observed for the anterior reach of the YBT among participants with a high weekly training volume, while the percentage of anterior reach of the YBT increased significantly among participants with a low training volume. To our knowledge, no studies have analyzed the influence of SFE on the kinematics of the foot arch and balance of trail runners. These findings suggest a positive effect of SFE in improving the AHI of the foot and dynamic balance in the anterior reach among trail runners; further, training volume influences the observed changes.

The abductor hallux is one of the largest intrinsic muscles of the foot, and it contributes to the supination of the midtarsal joint against the pronation force of the ground reaction. In other words, it stabilizes and even increases the medial longitudinal arch (Jung et al., 2011). Evidence shows that the IFM, such as the flexor digitorum brevis, quadratus plantae, and especially the abductor hallux, exhibit greater intrinsic activation in response to increased postural demands. Consequently, during single-leg support, there is greater IFM activation to stabilize and prevent the collapse of the MLA (Jung et al., 2011; Kelly et al., 2012). Our study observed improvements in AHI following SFE, which may reflect acute functional responses of the IFM, improved the AHI of the foot, which represents the kinematic changes of the MLA, in the anterior reach of the YBT among participants with high training volume. This finding is consistent with prior literature that supports an association between high chronic training loads and better performance and physical condition (Gabbett, 2016), including in individual sports like running (Scrimgeour et al., 1986), provided that the training load is appropriate (Gabbett, 2016). An optimal training load enhances performance while minimizing risks like fatigue and injury (Morton, 1997). Since IFM fatigue is linked to greater navicular drop (Headlee et al., 2008), individuals with higher training volumes, likely having better conditioned IFM, may have responded more effectively to the SFE. This could explain the observed improvements in AHI exclusively in this group. Postural control mechanisms, working in conjunction with muscle function, play a key role in stabilizing the foot arch during dynamic tasks. These findings highlight the complex interplay between neuromuscular and postural regulation in response to targeted SFE (Méndez-Rebolledo et al., 2015; Saavedra-Miranda & Mendez-Rebolledo, 2016).

Recent evidence reinforces the relevance of IFM not only in maintaining the MLA but also in contributing to dynamic postural control through their roles in proprioceptive feedback and neuromuscular coordination. In particular, the abductor hallucis, flexor digitorum brevis, and quadratus plantae act as both mechanical stabilizers and proprioceptive sensors, providing afferent input that is crucial for postural adjustment during dynamic tasks (Jastifer, 2023). In addition, it has been shown that the short foot exercise stimulates proprioceptors in the sole of the foot, increasing afferent input to the spinal cord, which in turn enhances voluntary muscle activation and stability (Newsham, 2010). This mechanism may partially account for the acute improvements observed in dynamic balance observed in all directions of the YBT following the SFE. A recent meta-analysis has examined the effects of structured and prolonged IFM training programs and demonstrated that such training significantly improves both dynamic postural balance and MLA morphology, highlighting its role in modulating plantar stiffness and force distribution during functional movements (Wei et al., 2022). While these findings support the relevance of IFM training, our results should not be interpreted in the same way, as we only observed acute, short-term changes. In this context, the activation of IFMs through the SFE may contribute to postural correction strategies relevant to trail running, where uneven terrain imposes high demands on proprioceptive acuity and foot stability (Pabón-Carrasco et al., 2020). Given that the AHI reflects the structural integrity of the MLA and may indirectly indicate intrinsic muscle engagement, future longitudinal studies should explore whether repeated use of the SFE could promote mechanical or neural adaptations. At present, however, our results only demonstrate acute, short-term functional effects.

On the other hand, when comparing according to training volume, only the subjects with low training volume showed significant improvements in the anterior reach of the YBT. These results contradict the previously described association of high loads with performance improvements (Gabbett, 2016). Our results can be interpreted based on the premise that individuals with lower weekly training volumes are less conditioned. Consequently, their initial adaptation to a functional test like the YBT may result in greater gains following training compared to those with more experience and higher weekly training volumes. For the latter group, the potential for improvement is smaller or occurs more gradually.

In the anterior reach of the YBT, the movement is performed primarily in the sagittal plane along the mediolateral axis. The medial longitudinal arch of the foot plays a crucial role in maintaining unipedal stability along this axis (Jung et al., 2011). The IFM are fundamental for single-leg balance, as their activity is strongly correlated with mediolateral sway (Kelly et al., 2012). In our study, improvements were observed in both foot arch kinematics and dynamic balance during the anterior reach task. While the low training volume group demonstrated improvements in the anterior reach direction of the YBT, this finding should be interpreted with caution. Although these participants had, on average, more years of trail running experience than those in the high-volume group, experience alone may not accurately reflect the neuromuscular conditioning required to respond acutely to targeted interventions. It has been demonstrated that runners with higher weekly mileage exhibit more efficient landing strategies and superior joint stiffness regulation (Verheul, Clansey & Lake, 2017), suggesting that current training volume may better represent neuromechanical adaptation than accumulated experience. Thus, it remains plausible that runners with higher recent training loads were better conditioned to exhibit neuromuscular changes in response to the short foot exercise.

Moreover, the anterior reach direction of the YBT is a complex motor task influenced by multiple factors, including lower limb strength, flexibility, trunk control, and motor learning. A weak association has been found between anterior reach performance and injury risk in elite youth athletes (Read et al., 2020), suggesting that this measure may not reliably reflect sensorimotor control or intrinsic foot function. Therefore, the improvement observed in the low-volume group may be attributed to non-specific motor adaptations, compensatory strategies, or prior familiarity with similar balance tasks, rather than to a direct effect of the SFE. As we did not assess joint kinematics, electromyographic activity, or proprioceptive acuity, we recommend interpreting this result with caution and encourage future research to incorporate more precise biomechanical and neuromuscular assessments to better clarify the mechanisms involved.

Limitations of this study include that the SFE was performed in a sitting position, which may have influenced muscle recruitment. Previous research suggests that performing the SFE in a standing position elicits a greater maximum voluntary contraction of the intrinsic foot muscles compared to the sitting position (Choi et al., 2021). Another important set of limitations includes the absence of a control group, lack of blinded assessment, and the non-randomized pre–post design, all of which limit internal validity and preclude strong causal inference. Similarly, the long-term effects of the SFE could not be assessed, and the sample size was relatively small. Therefore, our findings should be interpreted with caution and understood as preliminary evidence from a proof-of-concept study, intended to explore the feasibility of acute neuromuscular responses following a single SFE session. To address these issues, future research should employ randomized controlled trials with larger and more diverse cohorts to confirm these findings, ensure higher internal validity, and establish causal relationships.

This study opens a new line of research on trail runners. To our knowledge, it is the first study to measure the effect of the SFE on kinematics of the foot arch and postural control in this population. It provides relevant information for runners and sports personnel and indicates that the SFE may have potential as a pre-activation strategy to prepare the foot muscles for the perturbations and loads that trail running may present.

## Conclusion

This study suggests that a single session of the SFE may acutely improve the AHI of the foot in the anterior reach of the YBT in participants with high training volume, while runners with low training volume significantly improved their dynamic balance in the anterior reach. These preliminary findings indicate that the SFE has potential as an acute activation strategy for the IFM, but they should be interpreted with caution given the small sample size, the absence of a control group, and the exploratory nature of the design. Longitudinal randomized controlled trials are required to confirm these observations, determine their persistence over time, and evaluate their implications for performance and injury prevention in trail running.

##  Supplemental Information

10.7717/peerj.20364/supp-1 Supplemental Information 1 Raw data of Y-Balance Test and Arch Height Index

## Additional Information and Declarations

### Data Availability

The following information was supplied regarding data availability: The raw measurements are available in the Supplemental File.
